# Incidental cardiac findings on cardiovascular MRI obtained prior to atrial fibrillation ablation can affect patient management

**DOI:** 10.1186/1532-429X-16-S1-P144

**Published:** 2014-01-16

**Authors:** Akshay Pendyal, Duy T Nguyen, William Sauer, Robert A Quaife

**Affiliations:** 1 Internal Medicine, University of Colorado Anschutz Medical Campus, Aurora, Colorado, USA; 2 Cardiology, University of Colorado Anschutz Medical Campus, Aurora, Colorado, USA

## Background

Radiofrequency (RF) catheter ablation is an effective treatment for patients with symptomatic atrial fibrillation (AF). Cardiovascular MR (CMR) may be obtained pre-procedurally to assess pulmonary vein (PV) anatomy and be incorporated into electroanatomical mapping. Previous studies have reported the prevalence of incidental non-cardiac findings on CMR. Few, however, have documented the prevalence of incidental cardiac findings (ICFs) on clinical CMR. These findings represent clinically significant entities that can often alter ablation strategy and change patient management.

## Methods

204 patients underwent CMR prior to planned AF ablation. The indication for CMR was evaluation of PV anatomy prior to AF ablation. All studies were performed on a Siemens 1.5T scanner. Multiplanar oblique images were obtained using a single breath-hold true FISP gated cine technique. Perpendicular imaging of each pulmonary vein was performed using T2 dark blood imaging. Vein obstruction was assessed with phase contrast imaging. ICFs were considered if they arose from the heart, pericardium, coronary vessels, or great vessels. After classification of all ICFs, those that altered ablation strategy were then identified.

## Results

A total of 204 consecutive studies were analyzed. The mean age of the subjects was 60 ± 10.3 years. Male to female ratio was 2.4:1. A total of 39 ICFs were reported in 34 patients. This resulted in an overall prevalence of reported ICFs in the study of 19.1%, affecting 16.7% of patients (Table 1). Delayed contrast enhancement was the most common ICF encountered (6 patients). Pericardial disease was the 2nd-most common ICF encountered (5 patients). A total of 3 ICFs that altered ablation strategy were reported in 3 patients, forming 7.7% of ICFs and affecting 1.5% of patients (Table 2). Total/partial anomalous pulmonary venous return was the most common strategy-altering ICF encountered (2 patients). Unroofed coronary sinus (1 patient) was another. These unexpected ICFs resulted in an altered ablation strategy or referral to congenital heart disease specialists.

**Table 1:** 

Type of ICF	Patients (%)
Heavily trabeculated right atrium	1 (0.5%)

Hypertrophic obstructive cardiomyopathy	1 (0.5%)

Possible left atrial appendage clot	2 (1.0%)

Atrial septal aneurysm	5 (2.5%)

Delayed contrast enhancement	6 (2.9%)

Wall motion abnormality	4 (2.0%)

Patent foramen ovale	2 (1.0%)

Pericardial disease	5 (2.5%)

Aortic arch anomaly	3 (1.5%)

Apical-septal aneurysm	1 (0.5%)

Kink in distal aortic arch	1 (0.5%)

Unroofed coronary sinus	1 (0.5%)

Total/partial anomalous pulmonary venous return	4 (2.0%)

Atrial septal defect	1 (0.5%)

Subclavian artery anomaly	1 (0.5%)

**Table 2:** 

Type of ICF	Patients (%)
Total/partial anomalous pulmonary venous return	2 (1.0%)

Unroofed coronary sinus	1 (0.5%)

## Conclusions

While there are descriptions of non-cardiac findings on CMR obtained prior to AF ablation, there is a lack of data characterizing ICFs. Our findings indicate that ICFs are common and some can alter ablation strategy or change patient management. No specific guidelines regarding ICFs on CMR exist. As the present study demonstrates, it is critical for cardiac imaging specialists to recognize these occasionally clinically significant entities affecting patient management.

## Funding

None.

